# Predictors of Prolonged Hospital Length of Stay in Patients With Odontogenic Infections in Ghana

**DOI:** 10.1155/bmri/6612139

**Published:** 2026-06-27

**Authors:** Kwame Adu Okyere Boadu, Lydia Sarponmaa Asante, Paul Frimpong, Elijah Kwegyir Johnson, Victor Wireko Adu, Richard Okyere Boadu

**Affiliations:** ^1^ Department of Public Health, School of Public Health and Allied Sciences, Catholic University of Ghana, Fiapre, Ghana; ^2^ Sunyani Teaching Hospital, Sunyani, Ghana; ^3^ Department of Health Information Management, School of Allied Health Sciences, College of Health and Allied Health Sciences, University of Cape Coast, Cape Coast, Ghana, ucc.edu.gh

**Keywords:** abscess, cellulitis, Ludwig′s angina, necrotising fasciitis

## Abstract

**Background:**

Poor outcomes of odontogenic infections usually increase the length of stay (LOS) in hospitals, and the cost of treatment increases substantially. The LOS of patients with odontogenic infections is not set in stone. In clinical practice, it is observed that cost, certain medications and treatments, age and a plethora of factors influence this. However, it is unclear which factors have direct effects on it. As such, evidence‐based interventions become difficult.

**Methodology:**

The study utilised a retrospective observational approach and a total population sampling technique to investigate 286 out of the 811 patients admitted at the allied ward of STH from 2021 to 2025. Data was extracted from the Lightwave Health Information Management System and analysed with IBM SPSS 27, Claude (Anthropic, version Sonnet 4.6) and Python (Version 3.12).

**Results:**

A total of 286 patients were included, with a mean hospital length of stay (HLOS) of 9.28 ± 4.21 days. Necrotising fasciitis and Ludwig′s angina were associated with the longest admissions. On proportional odds ordinal logistic regression, severe infection classification (OR 25.39, 95% CI: 4.21–153.32) and necrotising fasciitis (OR 9.36, 95% CI: 3.87–22.61) were the strongest independent predictors of prolonged HLOS (all *p* < 0.001). HIV/AIDS, diabetes mellitus, hypertension, Ludwig′s angina and male sex were also significant independent predictors. The model demonstrated strong explanatory power (Nagelkerke *R* ^2^ = 0.686, *p* < 0.001). All predictor variance inflation factors were below 2.5, indicating no multicollinearity concerns.

**Conclusion:**

Infection severity, primary diagnosis, immunocompromising comorbidities and surgical interventions were the principal independent determinants of prolonged HLOS. Multispace involvement showed a crude association with extended HLOS but did not emerge as an independent predictor in the adjusted ordinal regression model. Early diagnosis and prompt, multidisciplinary management are crucial to reducing hospitalisation and improving patient outcomes.

## 1. Introduction

In resource‐constrained settings, access to dental care is not optimal, with a dentist population ratio of 1:10,000 [1]. This ratio implies that treatment is delayed and odontogenic infections (OIs), if diagnosed, deteriorate to fatalities. Inadequate advanced diagnostic use in incipient stages delays timely management. Severe OIs, including Ludwig′s angina, remain a significant clinical burden in sub‐Saharan Africa, with a rising incidence documented at major tertiary centres in Ghana [2]. The global burden of oral diseases, including dental caries and periodontal disease, disproportionately affects low‐ and middle‐income countries, where preventive services are inadequate [3].

Poor outcomes usually increase the length of stay (LOS) in the hospitals up to about 27.8 days [4], and the cost of treatment increases substantially. A comprehensive review of severe OI management spanning five decades highlighted that the median hospitalisation length ranges from 5 to 11 days and may extend to 60 days in complex presentations [5]. Interestingly, the LOS of patients with OIs is not set in stone. In clinical practice, it is observed that cost, certain medications and treatments, age and a plethora of factors influence this. However, it is unclear which factors have direct effects on it. As such, evidence‐based interventions become a hurdle to cross.

The impact of comorbidities on LOS will give a clear picture of the worrying comorbidities, so they can be targeted as well. Rural disparities can be addressed by investigating the role of socioeconomic factors on the hospital length of stay (HLOS) for infections. This will help the hospital to retool patient/bed utilisation policies for effective utilisation. For instance, if the average LOS for OIs is x days, the beds at the wards can be reserved for those days and anticipated to be used for those days when the infection patients are admitted. Hospital management can make informed procurement processes from key variables like HLOS. There is limited literature on this subject matter to make such informed decisions. Prior studies have identified the number of infected spaces and operative complexity as predictors of increased LOS in OIs [6, 7]. The role of inflammatory markers such as C‐reactive protein (CRP) in predicting hospitalisation duration has also been established [8, 9].

## 2. Methods

### 2.1. Study Area

This research was carried out in the allied ward, a ward designated for dental inpatients in Sunyani Teaching Hospital (STH), Sunyani. Sunyani East is the exact location of the hospital. Even though the hospital was built in 2003, it was not until November 2023 that it was converted to a tertiary facility [10].

### 2.2. Study Design and Type

A retrospective observational study was done for this study. Data of inpatients who were admitted at the allied ward from 2021 to 2025 on account of OIs were extracted from the Lightwave Health Information Management System (LHIMS) and ward admission register in April 2025.

### 2.3. Study Population

The study population involved all inpatients admitted to the allied ward of STH from January 2021 to January 2025. From the data, there were 811 admitted patients on account of variants of head and neck injuries and associated diseases.

### 2.4. Sampling Technique and Sample Size

#### 2.4.1. Sampling Procedure

A total population sampling technique was used in this study. The records of the 811 admitted persons were analysed to see which ones met the inclusion criteria. Per the study variables, records were extracted. However, neither a peculiar sampling nor exclusion of eligible cases was done. This was to ensure that every patient record meeting the inclusion criteria was well analysed and taken into consideration. In the long run, generalisability and adequate representation were achieved.

#### 2.4.2. Inclusion Criteria

i. Patients who are diagnosed with OIs.
ii. Patients admitted to the allied ward of STH from January 2021 to January 2025.
iii. Patients with complete hospital records.

#### 2.4.3. Exclusion Criteria

i. Patients who had more than 30% of their clinical records missing.

### 2.5. Sample Size Determination

The total number of records that were sampled was 286, via a total population sampling approach, from a population of 811 patient records. Of the 811, some inpatients were admitted because of head and neck cancers, head injuries, facial and scalp lacerations and various diseases of the eye, ear, nose and throat, as well as patients who were admitted to undergo various surgeries. The 286 patients represented patients who had OIs and met the inclusion criteria as stated supra.

### 2.6. Study Variables and Measurements

The dependent variable was HLOS, and it was measured as 1–3, 4–7, 8–11, 12–15 and > 16 days. Severe infection was classified using established clinical criteria at the time of hospital admission. A patient was categorised as having a severe infection if they presented with any of the following: Ludwig′s angina, necrotising fasciitis, descending mediastinal infection, involvement of four or more anatomical fascial spaces, signs of systemic sepsis, airway compromise requiring immediate intervention or requirement for intensive care unit (ICU) admission. All other cases were classified as nonsevere. This binary classification was included as a predictor variable in the regression models.

### 2.7. Data Collection Tool and Techniques

Data was extracted using an extraction tool with the number of days on admission, type of infection and total cost. The extraction tool was on both Google Forms and Microsoft Excel. About 30 records were extracted over a period of approximately 10 days for the total sample size to be realised.

### 2.8. Quality Control

Robust measures were enforced at every stage of the work, from pretesting of the extraction tool to completed extraction tool editing and data entry. This was to ensure data accuracy and correctness. Completed data were double‐checked before the next upload starts. At the end of every extraction day, data uploaded by Person A was cross‐checked by Person B and so forth, before the data for the day was secured on the cloud.

### 2.9. Data Entry and Processing

All data were exported from Google Forms to Excel for data cleaning. After cleaning the data, they were further saved and transferred to SPSS IBM Version 27 for analysis.

### 2.10. Data Analysis

The significant variables were then modelled to evaluate how they all influenced LOS using a reference of 1–3 days. Age was treated as a categorical variable grouped into clinically meaningful bands, and this grouping was informed by prior literature on OIs and clinical judgement, not by the outcome distribution. Predictor variables were selected a priori based on established clinical and epidemiological evidence, and all 11 predictors were entered simultaneously into the regression model without stepwise selection to minimise overfitting. The proportional odds assumption underlying the PO‐OLR was assessed using a Brant‐style visual test, examining the stability of log‐odds coefficients across ordinal thresholds; most predictors satisfied the assumption, with minor deviations noted for infection severity (Figure S5). Variance inflation factors (VIFs) were computed for all predictors; all VIFs were below 2.5, confirming no multicollinearity concerns (Figure S4).

The choice to employ four modelling approaches was motivated by methodological triangulation. With *n* = 286 patients and 11 predictors, the predictor‐to‐observation ratio of approximately 26:1 is adequate for ordinal logistic regression (a minimum of 10–15 observations per predictor is generally recommended). Ordinal logistic regression (PO‐OLR) and multinomial logistic regression (MLR) were designated as the primary analytical models given their direct interpretability and suitability for the ordered categorical outcome. Random forest (RF) and gradient boosting (GB) were included as sensitivity analyses to detect potential nonlinear associations and interactions not captured by regression; however, their substantial overfitting (in‐sample accuracy > 94% vs. fivefold cross‐validated accuracy < 79%) means their outputs are presented in the supporting information only and are not used to support primary inferential conclusions. The near‐complete separation observed in the multinomial model for some categories produced unstable coefficient estimates (very large values with *p* ≈ 1); these are noted explicitly in the Discussion and are not interpreted as effect sizes.

### 2.11. Use of Artificial Intelligence

Claude (Anthropic, version Sonnet 4.6) was used to assist with statistical computation during the data analysis and visualisation phases. Specifically, it was used to generate Python code for fitting the proportional odds ordinal logistic regression, MLR and ensemble machine learning models (RF and GB) using the scikit‐learn and statsmodels libraries. All model specifications, variable selection decisions, interpretation of results and clinical conclusions were determined exclusively by the authors. The AI tool did not independently make any analytical or clinical decisions; its role was strictly computational and programmatic under direct and continuous author supervision. IBM SPSS Version 27 was used for all descriptive statistics and chi‐square analyses. Python was used for the ordinal logistic regression and MLR and machine learning models because scikit‐learn and statsmodels provide ordinal regression and bootstrap confidence interval functionality not available in the version of SPSS used.

### 2.12. Ethical Considerations

The Catholic University of Ghana′s Ethical Review Board provided ethical clearance (CUG‐ERB‐122/25/4‐PG) for this study. The ethical clearance, together with the introductory letter from the department, was sent to the Acting Medical Director′s office of STH. It was also submitted to the Head of Department of the DEENT Sub‐Budget Management Committee (BMC). Confidentiality was assured and ensured throughout the study. Informed consent however was not sought as the data was secondary in nature.

## 3. Results

The LOS measured in days for patients admitted to STH due to OIs ranged from 2 to 16 days, with a mode of approximately 6 days and a standard deviation of more than 4 days. Thus, the average difference in the number of days spent by one patient from the other was 4 days. On average, a patient admitted to STH due to OIs spent approximately 9 days as an inpatient. As per the sample used for this study, the majority of the patients spent 4–7 days in the hospital on admission (83 [29.0%]), followed by 8–11 days (77 [26.9%]) and 12–15 days (74 [25.9%]). Only 26 (9.1%) of the patients sampled spent 16 days or more on admission.

As presented in Table 1, the majority of patients admitted with OIs had the infections affecting multiple spaces (70 [24.5%]), followed by those affected in the right submandibular area (65 [22.7%]), right buccal area (39 [13.6%]), left submandibular (35 [12.2%]) and submental area (24 [8.4%]). Only a few patients had infections affecting their sublingual area (4 [1.4%]). More than half of the patients sampled were active users of the National Health Insurance Scheme (NHIS) (200 [69.9%]), with 40 (14.0%) using private health insurance.

**Table 1:** Descriptive statistics on length of stay, specific affected spaces, insurance usage and immunocompromising conditions.

Factor	Frequency (*N* = 286)	Percentage (%)
LOS (Avg = 9.28; SD = 4.21)		
1–3 days	26	9.1
4–7 days	83	29.0
8–11 days	77	26.9
12–15 days	74	25.9
16 or more days	26	9.1
Specific spaces affected		
Left buccal	33	11.5
Right buccal	39	13.5
Left submandibular	35	12.2
Right submandibular	65	22.7
Sublingual	4	1.4
Submental	24	8.4
Multiple spaces	70	24.5
Others	16	5.6
Insurance used		
None	46	16.1
NHIS	200	69.9
Private insurance	40	14.0
Immunocompromising condition		
Diabetes mellitus	129	45.1
Hypertension	68	23.8
HIV	27	9.4
Underlying dental condition		
Dental caries	149	52.1
Dental trauma	10	3.5
Failed endodontic treatment	8	2.8
Periodontal diseases	89	31.1
Retained roots	30	10.5

*Note:* Source: Survey 2025.

Abbreviations: HIV, human immunodeficiency virus; LOS, length of stay.

Exactly 224 (78.3%) of the patients sampled had been diagnosed with immunocompromising conditions such as diabetes mellitus (DM) (129 [45.1%]), hypertension (HPT) (68 [23.8%]) and human immunodeficiency virus (HIV) (27 [9.4%]), as shown in Table 1. More than half of the patients diagnosed with OIs had dental caries as an underlying condition (149 [52.1%]), followed by periodontal diseases (89 [31.1%]) and retained roots (30 [10.5%]). Only a few of the patients had dental trauma or failed endodontic treatment (FET) as underlying conditions, with 10 (3.5%) and 8 (2.8%), respectively.

The chi‐square test of independence revealed that the LOS of a patient admitted to STH due to an OI was influenced by some sociodemographic factors. Amongst the factors that had significant influence on the LOS were age category of the patient (*p* < 0.001, *χ* ^2^ = 163.46, *φ* = 0.76), the primary diagnosis, thus the type of OI diagnosed of (*p* < 0.001, *χ* ^2^ = 154.37, *φ* = 0.74), the specific spaces affected by the infection (*p* < 0.001, *χ* ^2^ = 124.937, *φ* = 0.661), the type of insurance used (*p* < 0.001, *χ* ^2^ = 55.95, *φ* = 0.44), the type of immunocompromising conditions affected with (*p* < 0.001, *χ* ^2^ = 112.54, *φ* = 0.63) and the type underlying dental condition present (*p* < 0.005, *χ* ^2^ = 34.04, *φ* = 0.35). The phi (*φ*) measuring the effect sizes of the significant association that exists between the variable indicated a moderate to high strength of association ranging from 0.4 to 0.7, as shown in Table 2.

**Table 2:** Assessing the association between sociodemographic factors of patients and their respective length of stay in the hospital (*N* = 286).

Factors	*χ* ^2^(df)	*p*value (two‐tailed)	Effect size (*φ*)
Gender	2.49 (4)	0.647	0.09
Age category	163.46 (28)	< 0.001 ^∗∗^	0.76
Employment status	8.72 (4)	0.068	0.18
Weather season	6.44 (4)	0.169	0.15
Primary diagnosis	154.37 (12)	< 0.001 ^∗∗^	0.74
Specific spaces affected	124.94 (28)	< 0.001 ^∗∗^	0.66
Insurance used	55.95 (8)	< 0.001 ^∗∗^	0.44
Immunocompromising condition	112.54 (4)	< 0.001 ^∗∗^	0.63
Underlying dental condition	34.04 (16)	0.005 ^∗^	0.35

*Note:* Source: Survey 2025.

^∗^Significant at 5% alpha level;  ^∗∗^significant at 1% alpha level.

**Figure 1:**
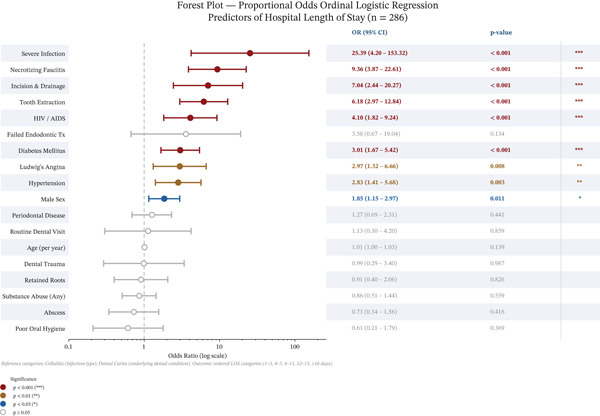
Forest plot of adjusted odds ratios (95% confidence intervals) from the proportional odds ordinal logistic regression model for predictors of hospital length of stay (*n* = 286). Filled circles denote statistically significant predictors; open circles denote nonsignificant predictors. Reference categories: cellulitis (infection type) and dental caries (underlying dental condition).

Figure 1 presents the fully adjusted odds ratios (ORs) and 95% confidence intervals from the proportional odds ordinal logistic regression model predicting HLOS across five ordered categories (1–3, 4–7, 8–11, 12–15 and ≥ 16 days) in 286 patients admitted with severe OIs. The overall model was statistically significant (likelihood ratio *χ* ^2^ = 301.76, df = 18, *p* < 0.001; Nagelkerke *R* ^2^ = 0.686), indicating that the predictors collectively explained a substantial proportion of the variance in HLOS.

The high Nagelkerke *R* ^2^ (0.686) is attributable to the inclusion of strongly discriminating clinical predictors (infection type and severity) and is consistent with values reported in studies using similar composite clinical predictor sets. VIFs for all predictors ranged from 1.02 to 2.11 (see Figure S4), confirming that multicollinearity does not account for model instability. The wide confidence intervals observed for the severity OR (4.21–153.32) reflect sparse cell counts in extreme HLOS categories rather than fundamental model failure and are consistent with the sample size. The proportional odds assumption was evaluated using a Brant‐style test comparing binary logistic coefficients across each HLOS threshold (Figure S5). Several predictors—notably infection severity, I&D, DM and HPT—demonstrated coefficient ranges exceeding 1.0 log‐odds across thresholds, suggesting partial departure from strict proportionality. Results should therefore be interpreted with this limitation in mind; the ordinal structure of the outcome is preserved, and inferential conclusions remain valid for the primary clinical questions addressed.

Infection severity was the factor most strongly associated with higher cumulative odds of being in a longer HLOS category across all thresholds (OR = 25.39, 95% CI: 4.20–153.32, *p* < 0.001), underscoring the decisive role of clinical acuity in determining duration of admission. In a proportional odds model, ORs represent the cumulative odds of being in a higher (more prolonged) HLOS category relative to all lower categories simultaneously. Amongst infection types, necrotising fasciitis was associated with substantially higher cumulative odds of being in a longer HLOS category relative to cellulitis (OR = 9.36, 95% CI: 3.87–22.61, *p* < 0.001), followed by Ludwig′s angina (OR = 2.97, 95% CI: 1.32–6.66, *p* = 0.008). Abscess was not independently associated with HLOS after full adjustment (OR = 0.73, *p* = 0.416).

Regarding surgical treatment, both incision and drainage (OR = 7.04, 95% CI: 2.44–20.27, *p* < 0.001) and tooth extraction (OR = 6.18, 95% CI: 2.97–12.84, *p* < 0.001) were significantly associated with longer hospitalisation. These associations should not be interpreted causally. Incision and drainage and tooth extraction are procedural requirements dictated by disease severity—they are performed precisely when infection has progressed to suppuration or when the offending tooth cannot be preserved. Their positive association with HLOS therefore reflects the greater severity of underlying disease at the time of decision‐making, rather than independently prolonging the hospital course. These variables function as severity surrogates within the model, consistent with the broader clinical literature [6, 7].

**Figure 2:**
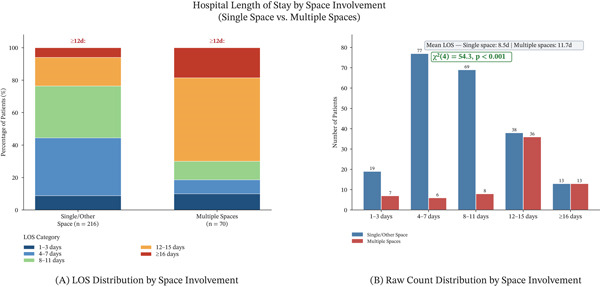
Hospital length of stay (HLOS) by anatomical space involvement. (A) LOS distribution by space involvement. Stacked percentage bar chart showing proportional distribution across five HLOS categories for single/other‐space (*n* = 216) and multiple‐space (*n* = 70) patients. The proportion of patients with HLOS ≥ 12 days was 22.7% for single‐space versus 70.0% for multispace involvement. (B) Raw count distribution by space involvement. Grouped bar chart of raw patient counts per HLOS category by space involvement. Patients with multispace infection had a significantly higher mean HLOS (11.7 vs. 8.5 days; *χ* ^2^ = 54.3, df = 4, *p* < 0.001).

Amongst immunocompromising comorbidities, HIV/AIDS (OR = 4.10, 95% CI: 1.82–9.24, *p* < 0.001), DM (OR = 3.01, 95% CI: 1.67–5.42, *p* < 0.001) and HPT (OR = 2.83, 95% CI: 1.41–5.68, *p* = 0.003) were each independently associated with prolonged HLOS after controlling for infection type, severity, treatment and all other covariates. Male sex was the only demographic predictor to attain statistical significance (OR = 1.85, 95% CI: 1.15–2.97, *p* = 0.011). Oral hygiene status, routine dental attendance, substance use history, age and underlying dental aetiology were not independently predictive of HLOS after adjustment (all *p* > 0.05). Age was not independently significant in the adjusted regression model (*p* = 0.139); however, it emerged as the single most important predictor in both machine learning models (RF importance: 0.201; GB: 0.190), suggesting a possible nonlinear or interaction‐mediated effect not fully captured by the proportional odds framework. Multispace involvement was significantly associated with prolonged HLOS (mean 11.7 vs. 8.5 days for single‐space involvement; *χ* ^2^ = 54.3, *p* < 0.001; Figure 2), with 70% of multispace patients experiencing HLOS ≥ 8 days compared with 41% of single‐space patients.

The proportional odds ordinal logistic regression was selected as the primary model for this analysis on the basis of three criteria: (i) It explicitly preserves the ordinal structure of the HLOS outcome, which multinomial regression does not, (ii) it yields directly interpretable ORs with 95% confidence intervals and *p* values suitable for clinical reporting and (iii) a Nagelkerke pseudo − *R* ^2^ of 0.686 indicates strong model fit by conventional thresholds. The ML models are presented as supplementary sensitivity analyses to corroborate variable importance rankings from the primary regression.

**Figure 3:**
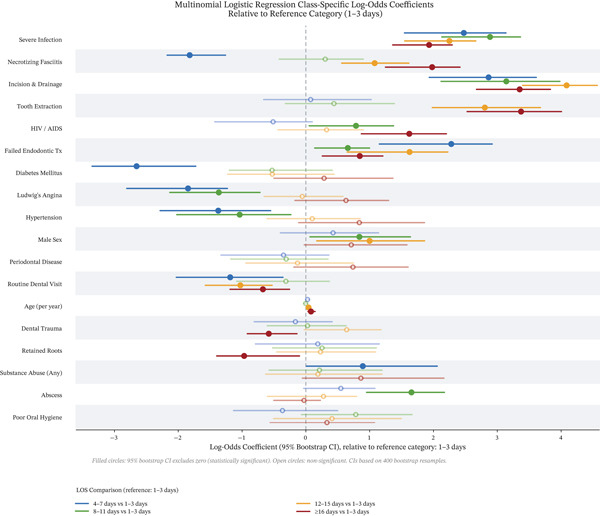
Class‐specific log‐odds coefficients from the multinomial logistic regression model, expressed relative to the reference category (1–3 days). Filled circles denote coefficients whose 95% bootstrap confidence interval (500 resamples) excluded zero; open circles denote nonsignificant coefficients.

Confidence intervals were derived from 500 bootstrap resamples; filled circles denote predictors whose 95% CI excluded zero (statistically significant), whereas open circles indicate nonsignificant associations. Coefficients for several categories exhibited extremely large standard errors and point estimates with *p* values approaching 1.0 (notably in the 12–15‐ and ≥ 16‐day strata), indicating near‐complete separation in sparse ordinal cells; these estimates are highly unstable and should not be interpreted as meaningful effect sizes (Figure 3).

Incision and drainage and tooth extraction consistently generated the largest positive log‐odds coefficients across the three longest LOS categories (12–15 and ≥ 16 days), with CIs lying entirely to the right of zero for the ≥ 16‐day comparison. Severe infection showed a marked positive gradient specifically for the 12–15‐ and ≥ 16‐day categories, with the coefficient for the ≥ 16‐day comparison being amongst the largest in the model.

A notable pattern was observed for DM and necrotising fasciitis: both showed large negative log‐odds for the 4–7‐day category relative to the 1–3‐day reference, reflecting that patients with these characteristics were systematically drawn away from the shortest LOS bands. HIV/AIDS and HPT displayed moderate positive coefficients for the longest HLOS categories, consistent with the ordinal logistic model findings. Across all predictors, the magnitude of the log‐odds coefficients increased monotonically from shorter to longer LOS categories, providing support for the proportional odds assumption underlying the primary model.

**Figure 4:**
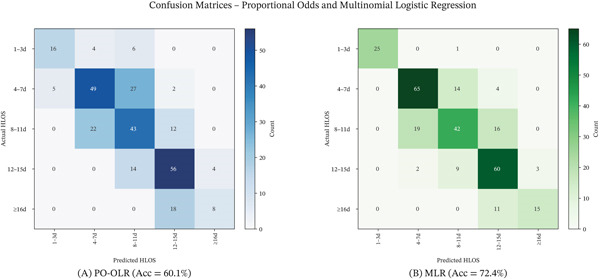
Confusion matrices for the proportional odds ordinal logistic regression (PO‐OLR) and multinomial logistic regression (MLR) models. Cells report raw counts of observed versus predicted HLOS categories; diagonal cells represent correct classifications. (A) PO‐OLR (Acc = 60.1*%*); (B) MLR (Acc = 72.4*%*).

Figure 4 presents row‐normalised classification heatmaps for the two primary models (PO‐OLR and MLR), showing the distribution of observed cases across predicted HLOS categories. Heatmap cells report raw counts; diagonal cells represent correct classifications. Heatmaps for the machine learning models (RF and GB) are presented in Figure S2.

The proportional odds ordinal logistic regression achieved an overall accuracy of 60.1% and a quadratic weighted kappa (QWK) of 0.791. The model performed best in the 4–7‐day (59% correct) and 12–15‐day (76% correct) categories, with greater misclassification at the distributional extremes (1–3 days: 62% correct; ≥ 16 days: 31% correct). The MLR achieved 72.4% accuracy (QWK = 0.860), with improved classification of the 4–7‐day (78% correct) and 1–3‐day (96% correct) categories, though the ≥ 16‐day category remained poorly captured (58% correct).

The RF and GB models achieved near‐perfect in‐sample accuracy (98.3% and 94.8%, respectively; QWK > 0.99 for both), with diagonal heatmap patterns indicating minimal misclassification on the training data. These results reflect overfitting to the training sample rather than genuine predictive generalisation and should be interpreted accordingly in conjunction with the cross‐validated accuracy estimates reported in Figure S1.

**Figure 5:**
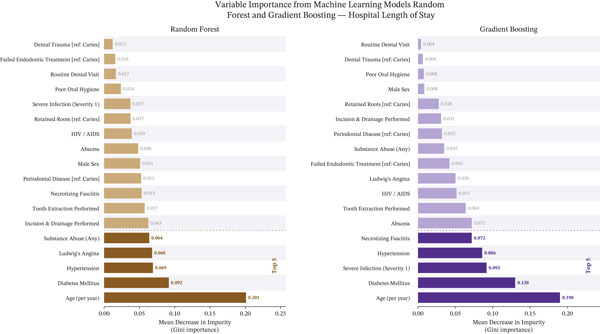
Variable importance (mean decrease in impurity, Gini importance) for the random forest and gradient boosting models. The five most important predictors in each model are highlighted. Age was the single most important predictor in both models (random forest 0.201; gradient boosting 0.190).

Figure 5 presents the mean decrease in Gini impurity (feature importance) for all 18 predictors from the RF and GB models, ranked in ascending order of importance. Darker bars with bold labels denote the top five predictors in each model; a dotted reference line marks the Top 5 threshold (the same variable importance rankings are also provided in Figure S3).

Both ML models consistently identified age as the single most important predictor of HLOS (RF: importance = 0.201; GB: 0.190), a finding not captured by the proportional odds regression, where age was nonsignificant after adjustment (*p* = 0.139). This discrepancy suggests that age may exert a nonlinear or interaction‐mediated influence on HLOS that the log‐linear proportional odds framework does not fully accommodate.

DM ranked second in both models (RF: 0.090; GB: 0.128), corroborating its significant independent association in the regression analysis. Infection severity (GB: 0.090), HPT (RF: 0.072; GB: 0.085) and Ludwig′s angina (RF: 0.067) completed the Top 5 rankings, broadly consistent with the primary regression findings. Notably, substance abuse ranked fifth in the RF model (importance = 0.068) which was higher than its position in the regression model, where it was nonsignificant. This suggests a possible interaction effect with other predictors that warrants further investigation. Oral hygiene behaviour and routine dental attendance ranked at the lowest end of variable importance in both ML models, consistent with their nonsignificance in the regression analysis.

**Figure 6:**
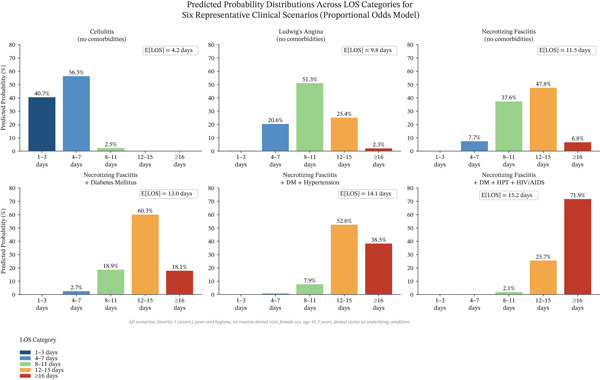
Predicted probability distributions across the five HLOS categories for six representative clinical scenarios, derived from the proportional odds model. Expected length of stay, E(LOS), is annotated for each scenario. All scenarios assume a 45.5‐year‐old female patient with severe infection, poor oral hygiene, no routine dental visit and dental caries as the underlying condition.

Figure 6 illustrates model‐derived predicted probability distributions across the five HLOS categories for six prototypical patient presentations of increasing clinical complexity, as generated by the proportional odds model. All scenarios assume a 45.5‐year‐old female patient with severe infection, poor oral hygiene, no routine dental visits and dental caries as the underlying dental condition. The expected length of stay (E[LOS]) is reported for each scenario, calculated as the probability‐weighted sum of category midpoints.

A patient presenting with cellulitis and no comorbidities had a predicted E(LOS) of 4.2 days, with probability concentrated in the 1–3‐day (40.7%) and 4–7‐day (56.5%) categories. In contrast, a patient with Ludwig′s angina and no comorbidities had a substantially higher E(LOS) of 9.8 days, with a modal probability in the 8–11‐day band (51.3%). Necrotising fasciitis without comorbidities yielded an E(LOS) of 11.5 days, predominantly distributed across the 12–15‐day category (47.8%).

Progressive accumulation of immunocompromising comorbidities in the setting of necrotising fasciitis produced a stepwise shift in predicted HLOS: The addition of DM raised E(LOS) to 13.0 days, with 60.3% probability in the 12–15‐day category; further addition of HPT increased E(LOS) to 14.1 days (38.5% in the ≥ 16‐day category) and concurrent HIV/AIDS elevated E(LOS) to 15.2 days, with 72.4% probability concentrated in the ≥ 16‐day category. These results demonstrate the multiplicative burden of comorbid immunosuppression on hospitalisation duration in patients with the most severe OIs.

**Figure 7:**
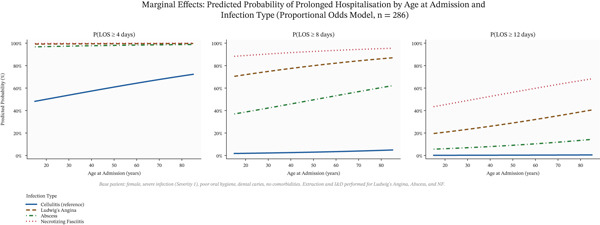
Marginal effects showing the predicted probability of prolonged hospitalisation as a function of age at admission and infection type, derived from the proportional odds model. (a) *p* (LOS ≥ 4 days). (b) *p* (LOS ≥ 8 days). (c) *p* (LOS ≥ 12 days). Base patient: female, severe infection, poor oral hygiene, dental caries, no comorbidities; extraction and incision and drainage performed for Ludwig′s angina, abscess and necrotising fasciitis.

Figure 7 presents model‐estimated marginal effect curves showing how the predicted probability of exceeding three HLOS thresholds—4, 8 and 12 days—varies continuously with patient age (15–85 years) across four infection types. Each curve holds all other covariates fixed at the base patient profile (female, severe infection, poor oral hygiene, dental caries and no comorbidities), isolating the joint effect of age and infection type on predicted outcome. Extraction and incision and drainage were included for Ludwig′s angina, abscess and necrotising fasciitis scenarios, but not for cellulitis.

For *p* (LOS ≥ 4 days) (Figure 7a), patients with necrotising fasciitis, Ludwig′s angina and abscess all approached or exceeded 97% probability regardless of age, reflecting the near‐universally prolonged admissions associated with these diagnoses. Cellulitis, by contrast, showed a probability rising from approximately 49% at Age 15 to 73% at Age 85, indicating that a meaningful proportion of younger cellulitis patients may be managed within 3 days.

For *p* (LOS ≥ 8 days) (Figure 7b), the four infection types diverged markedly. Necrotising fasciitis maintained a probability exceeding 88% across all ages, reaching 95% by Age 85. Ludwig′s angina demonstrated a steep age gradient (70% at Age 15, rising to 87% at Age 85), whereas abscess showed a more moderate gradient (38%–63%). Cellulitis remained substantially below the other infection types, with a probability between 2% and 6% across the age range, suggesting that hospitalisation beyond 1 week is uncommon in cellulitis patients without comorbidities.

For *p* (LOS ≥ 12 days) (Figure 7c), the separation between infection types was most pronounced. Necrotising fasciitis patients had a 45%–70% probability of staying 12 or more days across the age spectrum. Ludwig′s angina demonstrated an age‐dependent probability rising from 20% at Age 15 to 39% at Age 85. Abscess patients had a uniformly low probability (5%–13%), and cellulitis patients had a near‐zero probability (< 1%) of stays of 12 days or longer. These findings collectively demonstrate that infection type is the principal determinant of whether prolonged hospitalisation occurs, with age exerting a secondary but consistent modifying effect across all diagnoses.

## 4. Discussions

### 4.1. Age

Age was not an independent predictor of HLOS in the adjusted proportional odds regression model (*p* = 0.139), consistent with several prior studies in resource‐rich settings [11]. However, it ranked as the single most important variable in both the RF (importance = 0.201) and GB (importance = 0.190) models, suggesting that age exerts a nonlinear or interaction‐mediated influence that the log‐linear proportional odds framework does not fully capture. This discrepancy likely reflects age′s role as a proxy for comorbidity burden, frailty and delayed presentation rather than an independent biological driver of infection severity. The reason for this could be comorbidities or, perhaps, noted frailties in older people. In Sunyani, there is little to no special access and care for geriatrics. This might increase their LOS, as postoperative monitoring specific to their age group is not done. They are entitled to the one‐for‐all care given to all age cohorts.

In Gams et al.′s study, the results were in line with this (*p* = 0.011, *β* = 0.173), and their older patients had an extended HLOS. The study however attributed it to DM amongst the population [12]. A decade‐old study in a resource‐rich setting saw no correlation with age on HLOS (mean 41.5 years, *p* > 0.05). Maybe, there was a quick intervention for all age groups because of the adequately subsidised healthcare in that region, Lithuania [11]. The age effect from this study could also be attributed to limited resources for the elderly, making them stay in their homes for long before presenting to the hospital with their diseases well advanced.

### 4.2. Primary Diagnosis

The primary diagnosis of patients significantly influences their overall HLOS. In this study, Ludwig′s angina and necrotising fasciitis were independently associated with extended HLOS in the proportional odds ordinal logistic regression (*p* < 0.05), reflecting the highest clinical severity stratum and most resource‐intensive management pathways. The coefficient estimates from the MLR model for these diagnoses yielded extremely large values (indicative of near‐complete separation in sparse cells) and should not be interpreted as valid adjusted ORs; the ordinal model results are the primary reference. Per the practice in the OMFS unit in STH, broad‐spectrum antibiotics are administered, and airway management is initiated at first presentation. Whilst hospitalised, culture and sensitivity testing guides antibiotic refinement in the context of antimicrobial stewardship. Incision and drainage are maintained until output falls below 2 mL consistently before discharge.

For patients with NF, STH prefer to see wounds healed to a larger extent before they are let go. This convention was adopted by the OMFS team when it was realised the peripheral facilities around STH poorly managed wounds after discharge, leading to successive wound infections. Gams and his colleagues developed a severity scale for OIs. From their scale, a high score indicated severe infection, and this was associated with extended LOS (*p* < 0.001) [12]. Another study associated high LOS with the complexity of the infection (12.1 days for ≥ 4 spaces, *p* < 0.001). Patients in that study who had severe infections were initially managed in the ICU and transferred to the wards later, warranting a shorter LOS [11]. The system in STH is already constrained to follow this route of management. The result of this study underpins the constraints in managing OIs in resource‐constrained settings. Ludwig′s angina is described as a potentially fatal gangrenous cellulitis of the floor of the mouth and neck, whose management demands a trained multidisciplinary team for airway control, surgical debridement and broad‐spectrum antibiotics [13]. Necrotising fasciitis of odontogenic origin, when complicated by descent to the mediastinum, carries a mortality rate that underscores the need for early, aggressive surgical intervention [4, 5].

### 4.3. Specific Spaces Affected

Multispace involvement was a clinically and statistically significant determinant of prolonged HLOS in this cohort. Patients with multiple‐space involvement had a mean HLOS of 11.7 days compared with 8.5 days for single‐space cases (*χ* ^2^ = 54.3, df = 4, *p* < 0.001; Figure 2). Seventy percent of multispace patients experienced HLOS ≥ 8 days, versus 41% in the single‐space group, and 70.0% had HLOS ≥ 12 days compared with 22.7% for single/other‐space infections. Although multispace involvement did not emerge as an independently significant predictor in the final ordinal regression model after adjustment for infection type and severity—with which it is closely correlated—its bivariable association was strong (*χ* ^2^ = 124.94, *φ* = 0.66, *p* < 0.001; Table 2) and consistent with prior literature [11, 14]. In STH, it is not routine to take CT scans for every OI. This practice may perhaps lead to accurately diagnosing the affected spaces taking place later during treatment, invariably extending LOS. The role of physicoclinical management cannot be understated, as this has solutions for complicated cases. The gold standard however is a CT scan for all infection types to accurately predict involved spaces.

The 2015 study confirms this assertion with their multispace infection cases staying in the hospital for 12.1 days (*p* < 0.001). It is recommended that the hospital procure imaging tools like ultrasound guidance (USG) specific to the dental department to treat their cases. In doing so, LOS will be reduced; the hospital will have more bed spaces to admit overflow of cases from other wards and referral centres. Finally, the data on LOS should be well integrated in the hospital′s quality improvement programmes for effective monitoring. Cost‐effective treatment should be prioritised here. The paradox this presents is with respect to the recommendation of routine CT scans or USG for OI cases. Multispace infection, particularly involving the sublingual and submandibular spaces, has been independently associated with extended LOS and a higher risk of airway compromise in prior studies [14].

### 4.4. Immunocompromising Conditions

Comorbidities had a conflicting effect on HLOS in this study. Whilst the ordinal regression model demonstrated a crude association between immunocompromising comorbidities and extended HLOS, the crude OR for 4–7 days was 3.698 (*p* = 0.011), suggesting a detectable early association. The coefficient estimates from the multinomial model were highly unstable due to near‐complete separation (extremely large values with *p* ≈ 1) and are not interpreted as valid effect sizes. Glycated haemoglobin (Hb1Ac) is not routinely tested for admitted OI patients on the ward, which may lead to underdiagnosis of glycaemic dysregulation, ultimately attenuating statistical associations. In the same vein, aggressive treatment is applied to severe cases with immunocompromising conditions, which may curtail the most extended HLOS amongst the highest risk patients. Contrary to the present study, diabetes (12.4% prevalence) was statistically significant with LOS (*p* < 0.001, *β* = 0.380) in Gams et al. [12]. DM predisposes individuals to deep neck infections through impaired neutrophil chemotaxis and phagocytosis, and diabetic patients with deep neck infections have significantly longer hospital stays (19.7 vs. 10.2 days, *p* < 0.0001) and higher complication rates than nondiabetic counterparts [15]. A further retrospective study confirmed that abnormal blood glucose counts, even without a formal diabetes diagnosis, were independently associated with prolonged hospitalisation in patients with severe odontogenic abscesses [16].

DM was found in 7.6% of Heim et al.′s [16] study. Even though it had no significance on the patient′s LOS (*p* = 0.09), it was statistically associated with age (*p* = 0.049) and the spread of infection (*p* < 0.001). HIV infection similarly compounds the severity of odontogenic origin deep neck infections; HIV‐positive patients display a higher rate of odontogenic aetiology, greater risk of Ludwig′s angina‐related airway obstruction and a significantly elevated complication risk compared with immunocompetent patients [17]. Comorbidity screening should be routine for all OI cases. Instead of random blood sugar tests, the Hb1Ac test should be routine for all patients so that no one will be under‐ or misdiagnosed. If possible, CT scans should be routine for all cases of OIs, but with the glaring issue of resource constraints, USG of suspected affected spaces can be done. This will help with targeted drainage of pus from affected spaces.

The comorbidity prevalence observed in this cohort warrants contextualisation. The finding that 78.3% of admitted patients carried at least one immunocompromising condition, with DM present in 45.1%, substantially exceeds figures reported in comparable published series. Gams et al. [12] recorded a diabetes prevalence of 12.4% amongst OI inpatients, and Heim et al. [16] reported 7.6%, both in European tertiary centres. This disparity likely reflects several converging factors specific to the STH context: The hospital′s status as the sole tertiary referral facility for the Bono Region means that a disproportionate volume of medically complex and late‐presenting cases is channelled to this unit, enriching the cohort for high‐risk patients who have exhausted or bypassed lower level care. Beyond referral patterns, the broader epidemiological burden of HPT in Ghana, estimated national adult prevalence of approximately 10% Sanuade et al. [18], combined with low rates of diagnosis and glycaemic control in rural populations, plausibly contributes to a higher background comorbidity load amongst patients who develop severe OIs requiring hospitalisation. It is also important to acknowledge that the high comorbidity burden in this cohort may have acted as a confounding upstream variable: Immunocompromised patients are more likely to present late, to harbour polymicrobial infections and to develop multispace involvement, all of which independently drive infection severity and prolong HLOS. The ordinal regression model adjusted for comorbidities simultaneously with infection type and severity, attenuating but not eliminating the possibility of residual confounding between these closely interrelated variables. Readers should therefore interpret the comorbidity‐specific ORs in the context of this referral population, and caution is advised when extrapolating to settings with lower comorbidity prevalence.

### 4.5. Underlying Dental Conditions

Dental pathology influences the HLOS of patients with OIs. In the ordinal regression model, underlying dental condition (as a categorical group) did attain statistical significance at the bivariate level (*χ* ^2^ = 34.04, *p* = 0.005, *φ* = 0.35); however, no individual dental aetiology including periodontal disease or dental caries emerged as an independent predictor of HLOS after full adjustment in the proportional odds model (all *p* > 0.05). The MLR model produced coefficient and OR estimates for the ≥ 16‐day category that were extremely large and statistically unstable (*p* ≈ 0.94–0.95), consistent with near‐complete separation arising from sparse cell counts in that HLOS stratum; these values do not constitute valid effect sizes and are not interpreted here. As stated, the approach to dental care in this setting mimics a knee‐jerk reaction. As such, caries and periodontal diseases deteriorate to complex OIs, and management of these infections requires aggressive therapies that lead to an extended LOS. There was a synergistic effect with poor oral hygiene acting as a cofactor in Opitz et al. [19]. Dental caries and periodontal disease are the leading aetiologies of OIs globally, with the highest burden borne by low‐ and middle‐income countries in sub‐Saharan Africa, where access to preventive dental services remains limited [3]. Although one study inferred that dental pathologies had an impact on HLOS (*p* = 0.012) [12], most others did not find a correlation between HLOS and underlying dental pathology. Bali et al. [20] reported similar findings with no statistical backing, but added factors such as tooth impaction as potential mediators.

### 4.6. Insurance Status

The main nonclinical factor that affected the HLOS was insurance status. Patients without insurance, when compared with those with private insurance, showed an association with extended HLOS. The ordinal regression results suggest uninsured patients were more likely to fall into higher HLOS categories, consistent with the crude associations observed for shorter stay categories (crude OR for 4–7 days: 0.018, *p* = 0.018; crude OR for 8–11 days: 0.021, *p* = 0.050). The coefficient estimate from the multinomial model for the ≥ 16‐day category was extremely large and unstable (near‐complete separation) and should not be interpreted as a valid effect size. It can be inferred that patients without insurance delay hospital presentation and attempt alternative management until hospitalisation becomes unavoidable. Enrolment in Ghana′s NHIS has been shown to significantly increase utilisation of formal health services and reduce out‐of‐pocket expenditure, but approximately 48% of the population remains uninsured, sustaining financial barriers to timely presentation [21, 22].

Also, in STH, some patients who are initially discharged are made to stay on the wards for alternate wound dressing to be done for them, until they foot their bills and are finally discharged from the wards. Although 66.6% of the patients were uninsured in the study by Gams et al. [12] and the study linked the uninsured rate to treatment‐seeking behaviour, the respondents in the Lithuanian study had no problem whatsoever with access and early presentation to the hospital. Studies on the NHIS in Ghana consistently show that insured individuals are more likely to choose formal healthcare providers and less likely to self‐medicate than their uninsured counterparts. Antibiotic stewardship remains a critical concern in the management of OIs, given that approximately 10% of all antibiotic prescriptions globally originate from dental practice and up to 80% of these are deemed inappropriate, contributing to antimicrobial resistance [23].

### 4.7. Study Limitations

This study has some limitations that should be considered when interpreting the findings. First, the retrospective single‐centre design at STH limits the generalisability of results to other healthcare settings; the high Nagelkerke *R* ^2^ (0.686) likely reflects the clinical homogeneity of this population rather than universal predictive power, and external validation in multicentre or national cohorts is warranted. Second, the small number of patients at the distributional extremes of HLOS—particularly those with severe classification who had short admissions—contributes to the wide confidence intervals observed for some predictors (notably OR = 25.39, 95% CI: 4.20–153.32). Finally, unmeasured confounders such as antibiotic regimen, time‐to‐surgery interval and clinician‐level factors could not be accounted for in the current analysis.

## 5. Conclusion

OIs impose a considerable hospitalisation burden, with infection type, disease severity and immunocompromising comorbidities emerging as the principal determinants of prolonged HLOS. Necrotising fasciitis and Ludwig′s angina carry the greatest risk of extended admission, and the coexistence of DM, HPT or HIV/AIDS substantially amplifies this risk. The requirement for incision and drainage or tooth extraction was associated with longer HLOS; these associations reflect disease severity at the time of presentation rather than a causal effect of the procedures themselves and should be interpreted as severity markers within the model. Multispace involvement showed a crude association with extended HLOS but did not emerge as an independent predictor in the adjusted ordinal regression model and should not be cited as a standalone independent risk factor. These findings underscore the importance of early diagnosis, prompt surgical management and optimisation of underlying systemic conditions in reducing hospitalisation duration. Strengthening preventive dental services and improving access to routine oral healthcare may mitigate the upstream factors that drive disease progression to severe, hospitalisation‐requiring presentations. Clinicians and health systems managing OIs should recognise the compounding effects of comorbidity burden, infection type and severity when anticipating resource utilisation and planning patient care pathways.

### 5.1. Recommendations

This study highlights the significant association between HLOS and the severity of OIs. We recommend strengthening early referral systems, enhancing public awareness of the dangers of untreated dental infections and developing standardised multidisciplinary management protocols. Additionally, investments in preventive dental care and hospital infrastructure, coupled with antibiotic stewardship, are essential to reducing prolonged hospital stays and improving patient outcomes.

## Author Contributions

Kwame Adu Okyere Boadu: conceptualisation, investigation, methodology, writing—original draft, writing—review and editing, formal analysis and data curation; Lydia Sarponmaa Asante: supervision, writing—review and editing, project administration and methodology; Paul Frimpong: resources and writing—review and editing; Elijah Kwegyir Johnson: software, validation and visualisation; Victor Wireko Adu: visualisation, data analysis and writing—original draft; Richard Okyere Boadu: methodology, software, validation and visualisation.

## Funding

No funding was received for this manuscript.

## Disclosure

All authors have read and approved the final version of the manuscript. Kwame Adu Okyere Boadu had full access to all the data in this study and takes complete responsibility for the integrity of the data and the accuracy of the analysis. Kwame Adu Okyere Boadu affirms that this manuscript is an honest, accurate and transparent account of the study being reported, that no important aspects of the study have been omitted and that any discrepancies from the study as planned have been explained.

## Conflicts of Interest

The authors declare no conflicts of interest.

## Supporting information


**Supporting Information** Additional supporting information can be found online in the Supporting Information section. The supporting information accompanying this article contains five figures. Figure S1: The in‐sample and fivefold cross‐validated accuracy of the random forest and gradient boosting models, illustrating the degree of overfitting. Figure S2: The in‐sample confusion matrices for these two machine learning models. Figure S3: The variable importance rankings (mean decrease in impurity) for the random forest and gradient boosting models. Figure S4: The multicollinearity assessment, comprising the pairwise correlation matrix and variance inflation factors for all predictors. Figure S5: The Brant‐style visual assessment of the proportional odds assumption across hospital length of stay thresholds. These figures (Figures S1–S5) are referenced at the relevant points in the Results and Discussion sections.

## Data Availability

The data that support the findings of this study are available from the corresponding author upon reasonable request.
