# Supplementary material for: Predictors of Prolonged Hospital Length of Stay in Patients With Odontogenic Infections in Ghana
Source: Biomed Res Int. 2026 Jun 27;2026:6612139. doi: 10.1155/bmri/6612139 (PMC13309896; doi:10.1155/bmri/6612139)

**SUPPLEMENTARY MATERIALS**

Supplementary Figure S1. Machine Learning Model Accuracy (RF and GB — In-Sample vs Cross-Validated). Bar chart comparing in-sample training accuracy and 5-fold stratified cross-validated accuracy for the Random Forest (RF) and Gradient Boosting (GB) models. The large gap between training and CV accuracy (>19 percentage points) indicates substantial overfitting; these models are presented in supplementary only and their outputs are not used to support primary inferential conclusions.


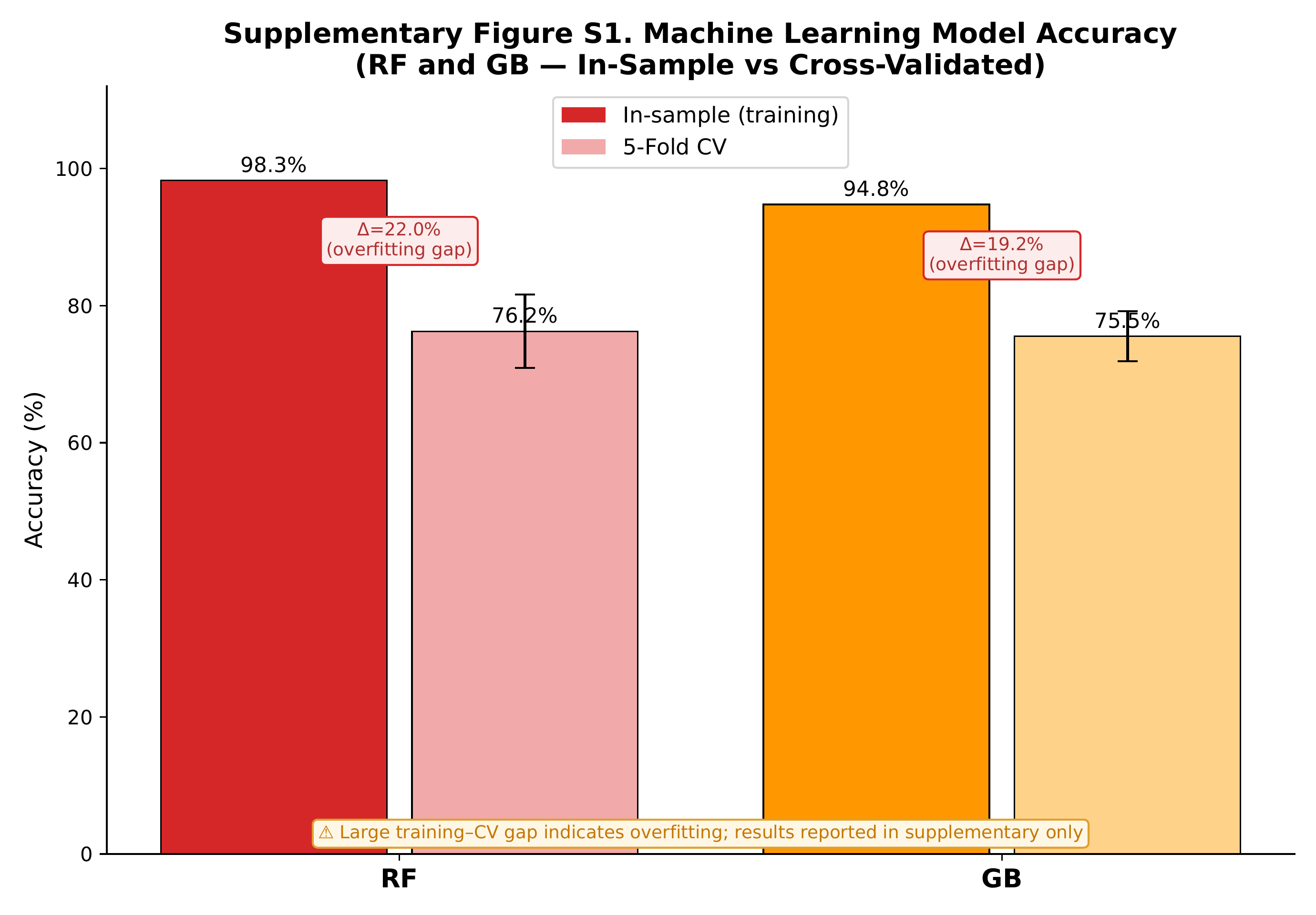


Supplementary Figure S2. Classification Heatmaps — Random Forest and Gradient Boosting. Row-normalised confusion matrices for RF (panel A) and GB (panel B) showing the distribution of observed cases across predicted HLOS categories. Overfitting warning banners emphasise that these results should not be used for primary inference.


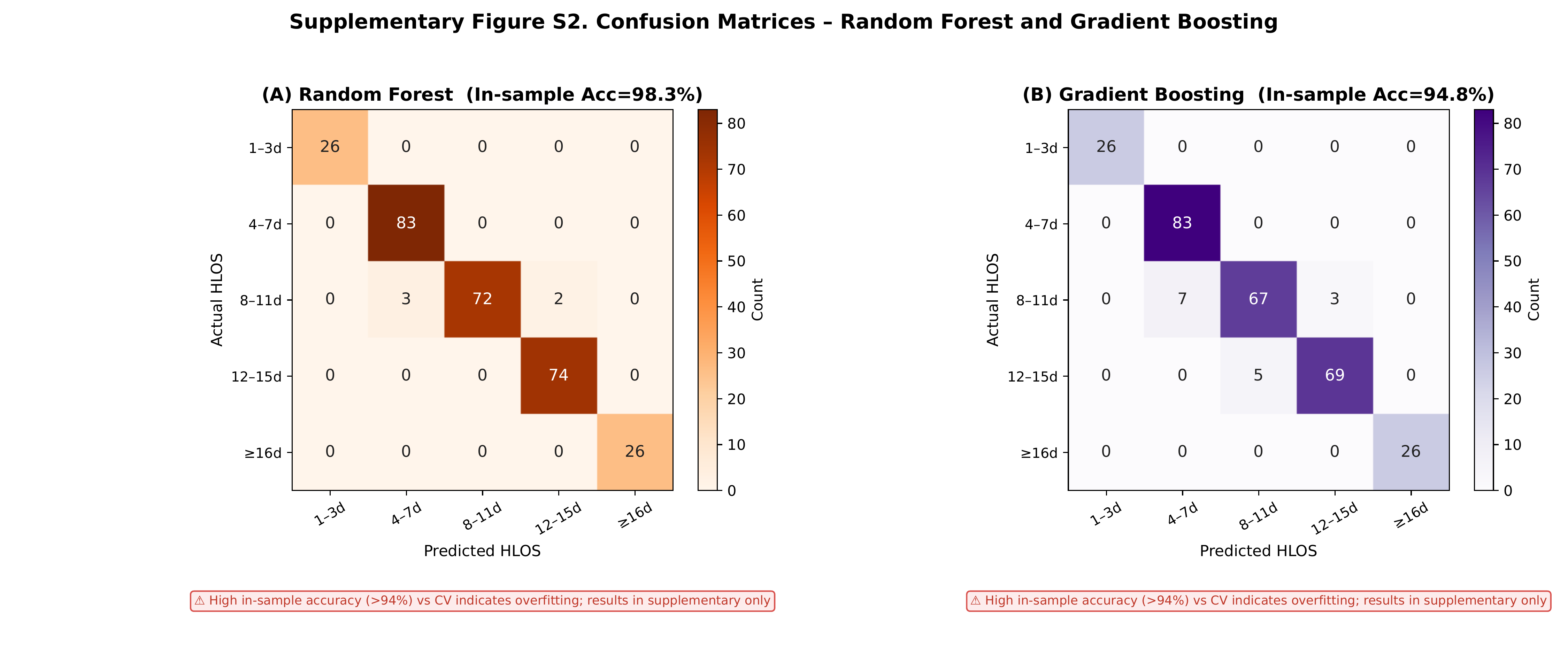


Supplementary Figure S3. Feature Importance Rankings from Machine Learning Models. Mean decrease in Gini impurity for all 18 predictors from RF and GB models, ranked in ascending order. Both models identified age as the most influential predictor. Results are presented as supplementary only due to the models' overfitting concerns.


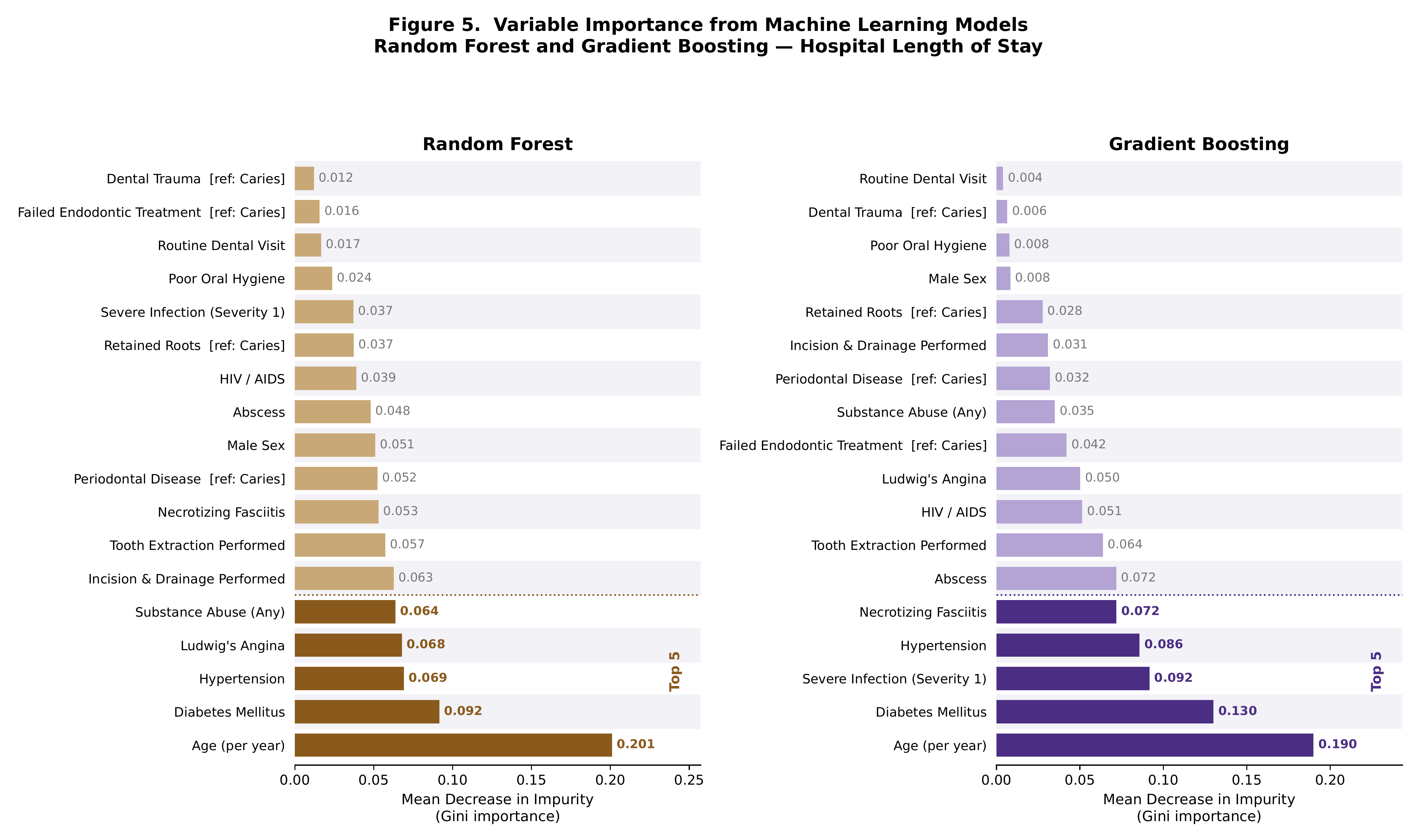


Supplementary Figure S4. Multicollinearity Assessment (formerly Supplementary Figure A). (A) Pairwise Pearson correlation matrix for the 11 regression predictors; strongest correlation r = 0.62 between NF and Ludwig’s angina. (B) Variance inflation factors (VIFs), all ranging from 1.02 to 2.11, well below the threshold of 2.5, confirming no multicollinearity concerns.


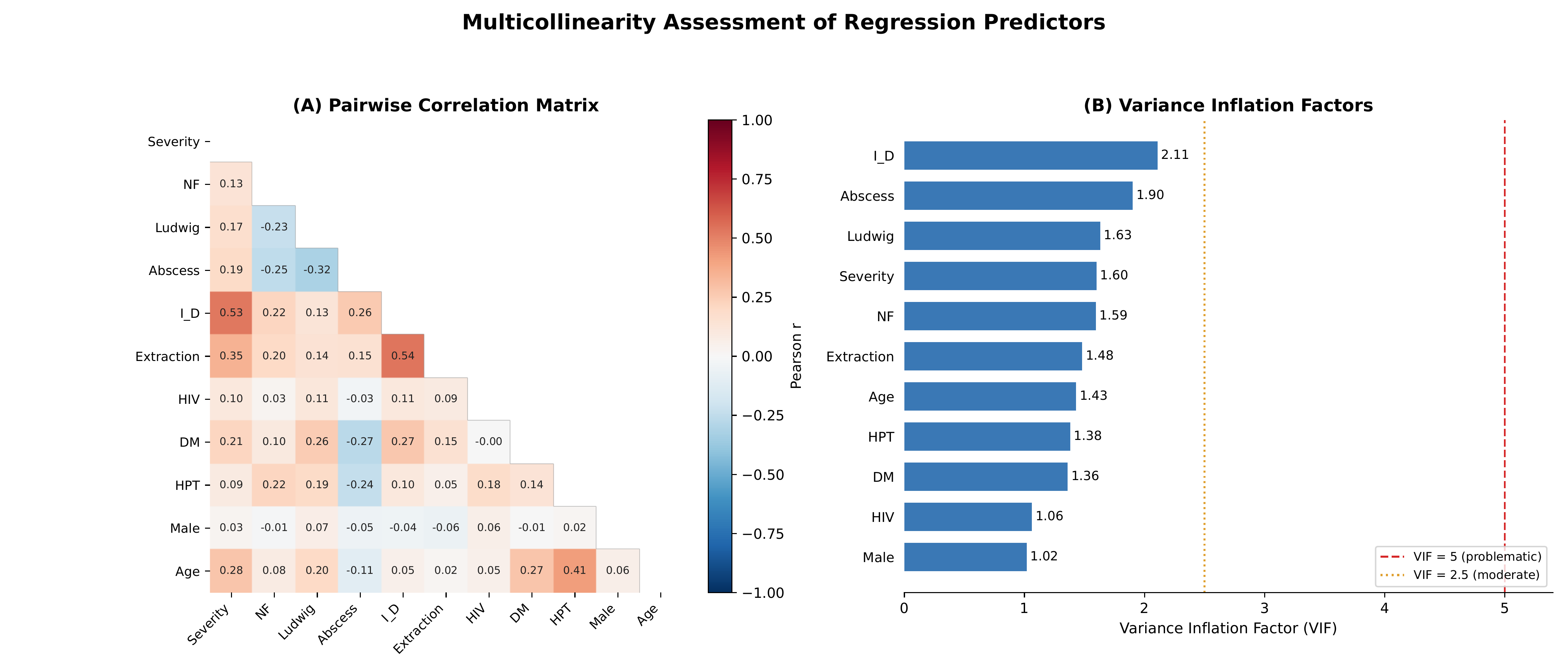


Supplementary Figure S5. Proportional Odds Assumption — Brant-Style Visual Test. Log-odds coefficient estimates plotted at three ordinal thresholds. Most predictors demonstrate stable coefficients across thresholds, supporting the proportional odds assumption. Minor deviations for infection severity are noted but do not invalidate the model.


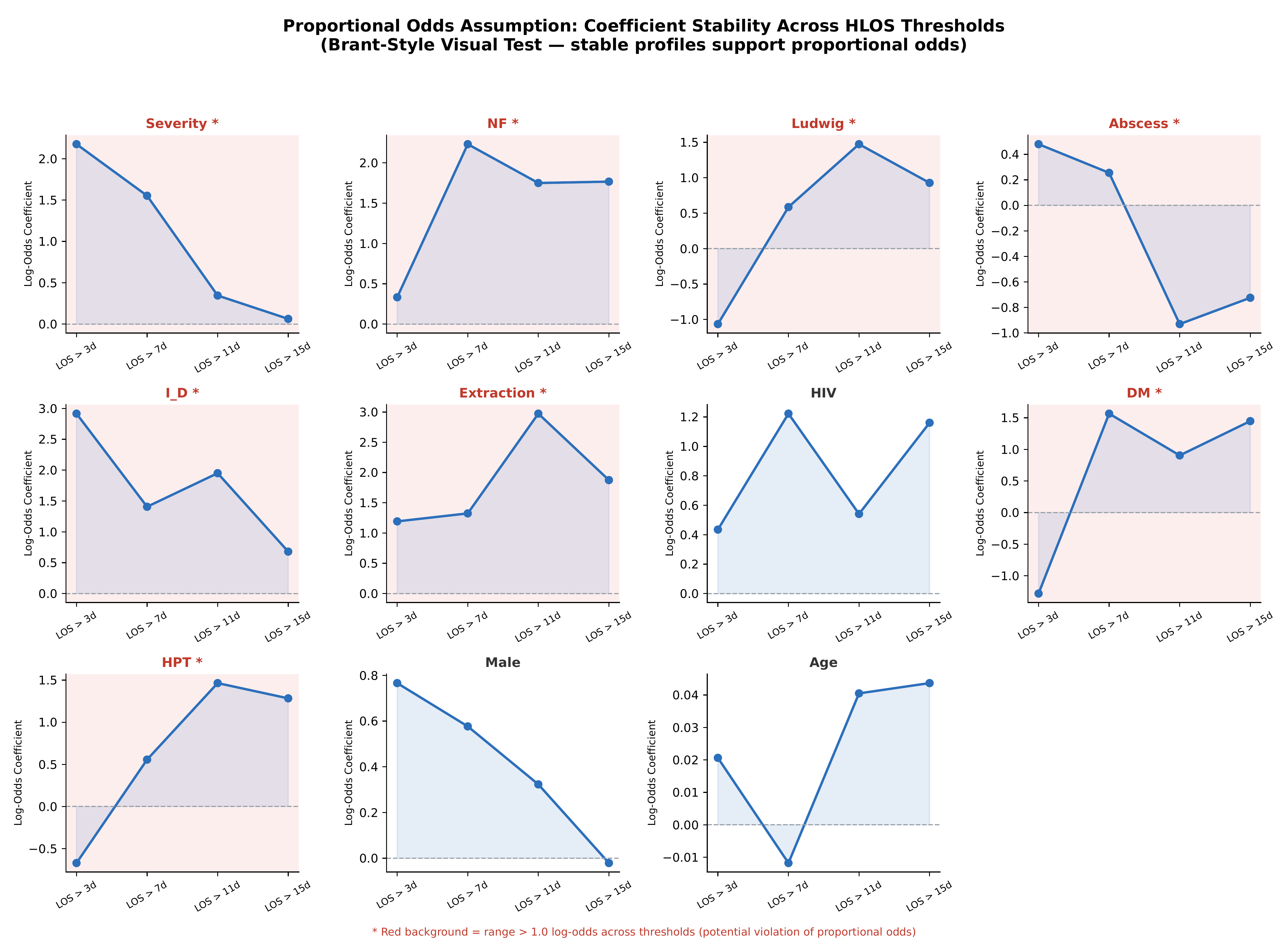

Supplement: Supplementary file 1 — Supporting Information Additional supporting information can be found online in the Supporting Information section. The supporting information accompanying this article contains five figures. Figure S1: The in‐sample and fivefold cross‐validated accuracy of the random forest and gradient boosting models, illustrating the degree of overfitting. Figure S2: The in‐sample confusion matrices for these two machine learning models. Figure S3: The variable importance rankings (mean decrease in impurity) for the random forest and gradient boosting models. Figure S4: The multicollinearity assessment, comprising the pairwise correlation matrix and variance inflation factors for all predictors. Figure S5: The Brant‐style visual assessment of the proportional odds assumption across hospital length of stay thresholds. These figures (Figures S1–S5) are referenced at the relevant points in the Results and Discussion sections. [file BMRI-2026-6612139-s001.docx]
